# Subcortical origin of nonlinear sound encoding in auditory cortex

**DOI:** 10.1016/j.cub.2024.06.057

**Published:** 2024-08-05

**Authors:** Michael Lohse, Andrew J. King, Ben D.B. Willmore

**Affiliations:** 1 Sainsbury Wellcome Centre for Neural Circuits and Behaviour, University College London, London W1T 4JG, UK; 2 Department of Physiology, Anatomy, and Genetics, University of Oxford, Oxford OX1 3PT, UK

**Keywords:** hierarchical organization, population communication, inferior colliculus, midbrain, medial geniculate body, thalamus, auditory cortex, descending projection, optogenetics, nonlinear

## Abstract

A major challenge in neuroscience is to understand how neural representations of sensory information are transformed by the network of ascending and descending connections in each sensory system. By recording from neurons at several levels of the auditory pathway, we show that much of the nonlinear encoding of complex sounds in auditory cortex can be explained by transformations in the midbrain and thalamus. Modeling cortical neurons in terms of their inputs across these subcortical populations enables their responses to be predicted with unprecedented accuracy. By contrast, subcortical responses cannot be predicted from descending cortical inputs, indicating that ascending transformations are irreversible, resulting in increasingly lossy, higher-order representations across the auditory pathway. Rather, auditory cortex selectively modulates the nonlinear aspects of thalamic auditory responses and the functional coupling between subcortical neurons without affecting the linear encoding of sound. These findings reveal the fundamental role of subcortical transformations in shaping cortical responses.

## Highlights

• Subcortical transformations largely explain nonlinear encoding in auditory cortex
• Population communication models substantially outperform receptive field models
• Feedforward transformations of sound encoding are irreversible and lossy
• Cortex selectively modulates nonlinear responses and functional coupling in thalamus

## Graphical abstract

**Figure:**
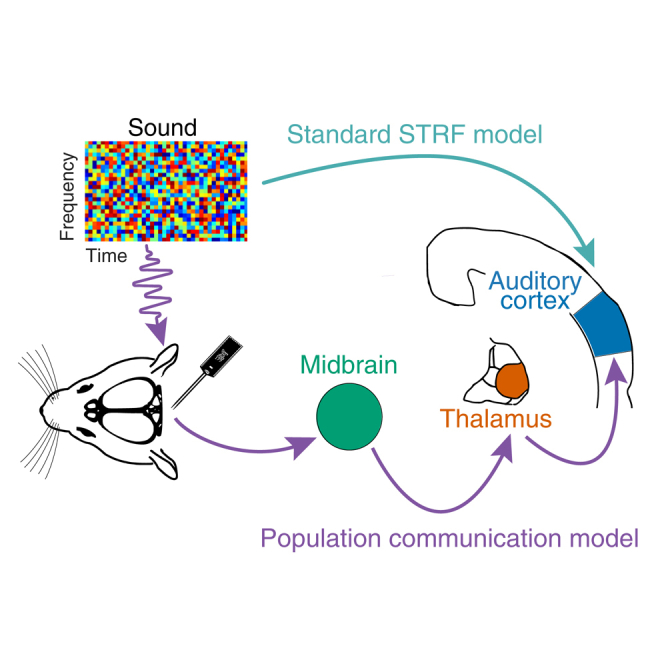

## Teaser

Lohse et al. show that higher-order representations of sound in the auditory cortex are largely explained by subcortical transformations of auditory information. A series of irreversible transformations take place in the ascending auditory pathway, and the cortex feeds back signals that modulate nonlinear responses and functional coupling in the thalamus.

## Introduction

Sensory systems need to represent the external environment in ways that allow animals to use this information for survival. The dominant tools for characterizing the tuning properties of individual sensory neurons are spatiotemporal (visual) and spectrotemporal (auditory) receptive fields (STRFs). These models provide a linear mapping from the spatio/spectrotemporal variables in the environment to neuronal activity and, with an output nonlinearity, are reasonably successful at describing and predicting neuronal responses at lower levels of the visual and auditory pathways. For example, measuring STRFs provides an effective way of characterizing the tuning properties of auditory nerve fibers.^1^

At higher levels of the sensory pathways, however, neural representations are seemingly more abstract and correspondingly harder to model.^2^^,^^3^ It is well established that STRF models perform relatively poorly in sensory cortex, most likely because cortical coding takes place in complex networks of highly interconnected excitatory and inhibitory cells.^4^^,^^5^^,^^6^ However, it has proven challenging to produce nonlinear models that capture cortical sensory processing as accurately as STRF models capture the behavior of subcortical neurons. Moreover, and particularly in the auditory system, it is not known to what degree the coding of complex stimuli in the cortex reflects subcortical transformations of the input.

To better understand how auditory neurons represent the environment, much effort has been made to expand STRF models—for example, by incorporating sensory and behavioral contexts^7^^,^^8^^,^^9^^,^^10^^,^^11^^,^^12^^,^^13^^,^^14^ as well as by exploring different nonlinearities.^9^^,^^15^^,^^16^^,^^17^^,^^18^ Although these approaches have provided insights into what features sensory neurons may represent, they have yet to result in a generally accepted model of how cortical auditory representations differ from subcortical representations or how and where neuronal representations of sounds in the auditory pathway are nonlinearly transformed into the complex representations found in cortex. Although cortical neurons are less able than subcortical neurons to follow rapidly varying sounds,^19^^,^^20^ the shapes of the STRFs along the ascending auditory pathway are remarkably similar. This is in marked contrast to the qualitative transformations that take place in the visual system.^21^^,^^22^^,^^23^

Another challenge is posed by the difficulty of fitting ever more complex models to limited physiological datasets. As the complexity of the models increases, the capacity of a limited dataset to constrain these models precisely decreases, resulting in suboptimal predictions. As a result, models of auditory cortical neurons fail to capture much of their stimulus-dependent variability. It is not clear to what degree this arises from nonlinear processing, variability in the responses, or imprecision in cortical encoding.

The cortex returns descending projections to nearly all subcortical levels, including the thalamus and midbrain. The excitability and response properties of neurons at different subcortical levels can be altered by manipulating the activity of neurons in the auditory cortex (reviewed in Suga,^24^ Bajo and King,^25^ and Souffi et al.^26^), and there is growing evidence that these descending corticofugal projections play important roles in auditory perception^27^^,^^28^ and learning.^29^ However, it is not yet known how these projections contribute to linear or nonlinear subcortical sensory encoding.

Here, we investigate the origin of nonlinear representations of sound in the auditory cortex. By exploring the nature of ascending and descending population communication between the auditory midbrain, thalamus, and cortex, we show that nonlinear subcortical transformations profoundly shape the response properties of cortical neurons. By combining this approach with optogenetic manipulation of cortex, we further demonstrate that cortex selectively modulates the nonlinear representations in thalamus and the functional coupling between subcortical neurons without affecting the linear encoding of sound. Furthermore, this new population communication model (PCM) allows us to predict to an unprecedented degree the sound-evoked responses of cortical neurons.

## Results

**Figure 1:**
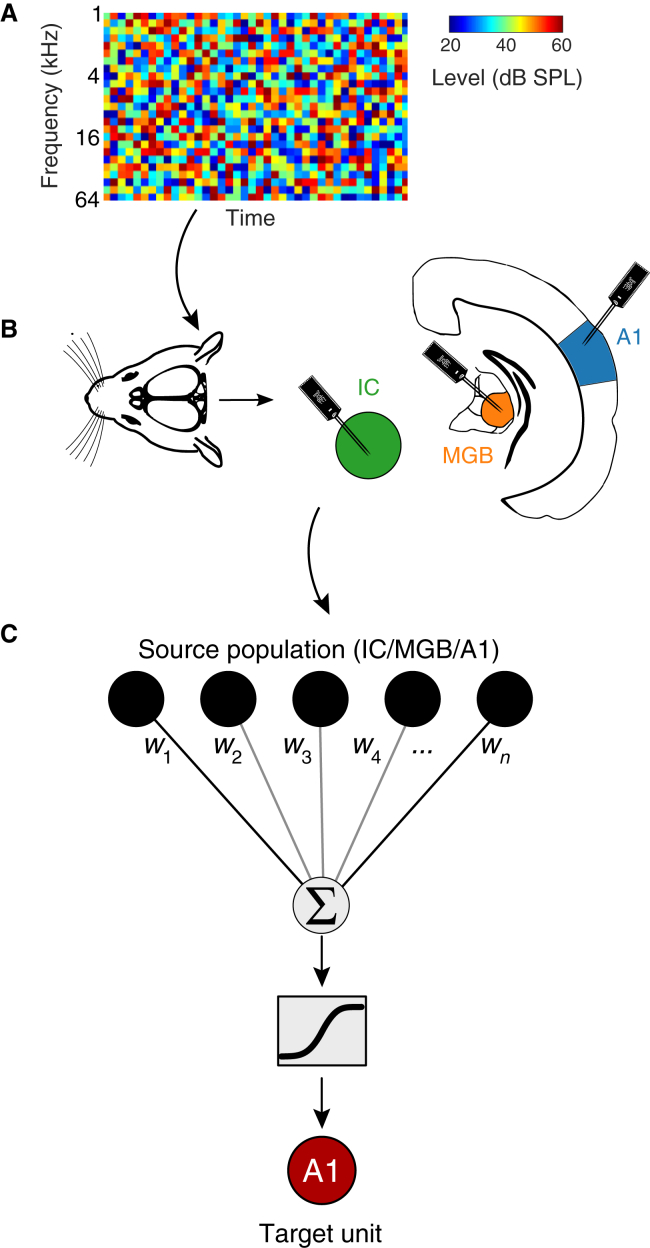
Population communication models (A) Dynamic random chords were presented to awake and anesthetized mice. (B) Electrophysiological recordings were made from inferior colliculus (IC), the medial geniculate body of the thalamus (MGB), and primary auditory cortex (A1). (C) Population communication models (PCMs) were used to explore communication between populations of neurons. Neurons in one or more areas (the source population) are used as input to the model, which is trained to describe the responses of each neuron (target unit) in the dataset. See also Figure S1.

To understand how the encoding of sound in auditory cortex arises as a function of inputs from populations of other auditory neurons, we recorded extracellular responses from 1,403 units (from 31 mice) to identical complex sounds at three levels of the auditory system: the midbrain (inferior colliculus [principally the central nucleus], IC, *n* = 432), thalamus (medial geniculate body [principally the ventral division], MGB, *n* = 355), and primary auditory cortex (A1, *n* = 616). We developed a PCM, which describes the stimulus-dependent activity of individual units in terms of the stimulus-dependent activity of populations of other units in the auditory system (Figure 1). The PCM is a linear-nonlinear encoding model, involving regularized linear regression between the response of the target unit and a set of input signals at multiple time lags, followed by a static nonlinearity (STAR Methods). Thus, the summation of inputs in this model is purely linear, but simple neuronal output nonlinearities, such as thresholds and saturation, can be accounted for by the static nonlinearity. In these respects, PCMs are similar to classical linear-nonlinear STRF models (Figure S1). However, in PCMs, the input signals are the recorded responses of other units rather than the sound itself, enabling us to directly probe the transformations of information that occur between the input units and the target unit.

### Subcortical transformations account for nonlinear auditory cortical responses

**Figure 2:**
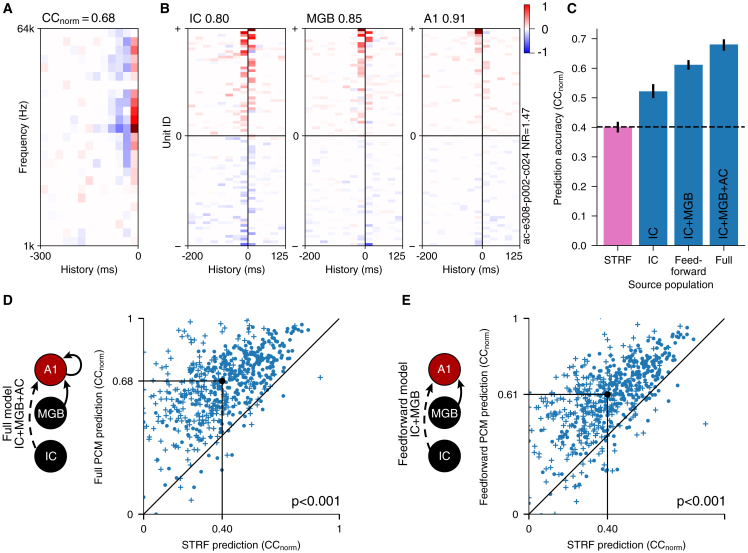
Feedforward population communication greatly improves the predictability of auditory cortical responses compared with receptive field models (A) Example of an STRF from a unit in A1. (B) Examples of PCM weights that describe the responses of the same A1 unit as in (A). Each set of PCM weights describes an optimized linear mapping between the responses of the source population and the responses of the target unit. (C) Population communication models (blue bars) greatly outperform STRF models (pink bar), and the predictability of auditory cortical increases as more nonlinear transformations across the ascending pathway are included (*p* < 0.001; *n* = 616 units). Error bars are 95% nonparametric confidence intervals. (D) Comparison of the prediction performance of auditory cortical units between the full PCM (i.e., using populations from IC, MGB, and A1 to capture the variability of auditory cortical neurons) and the STRF (*p* < 0.001; *n* = 616 units). Black dot indicates medians. (E) As in (D), but comparing feedforward PCM predictions (i.e., using populations from IC and MGB to capture the variability of auditory cortical neurons). Plusses denote units recorded under anesthesia, and filled circles denote units recorded in awake animals. See also Figure S2.

We first asked whether the PCM could accurately capture the responses of auditory neurons at higher levels of the auditory pathway. To do this, we trained the model to describe the responses of each recorded unit in A1 in terms of the mean responses of all non-simultaneously recorded units (enabling us to focus on stimulus-dependent activity, excluding any contribution from noise correlations between simultaneously recorded units). We also trained a standard linear-nonlinear STRF model (Figure 2A) and a network receptive field (NRF) model^18^ on the same units for comparison. We compared the ability of the models to predict real neuronal responses by measuring the normalized correlation coefficient (CC_norm_^30^) on a held-out dataset.

We found that a PCM predicting cortical neural activity from populations of units in IC, MGB, and A1 (“full” model) substantially outperformed the STRF model. Specifically, the PCM captures 69.4% (CC_norm_^IC+MGB+A1^/CC_norm_^STRF^: 0.68/0.40) and 76.4% (CC_norm_^IC+MGB+A1^/CC_norm_^NRF^: 0.68/0.38) more of the variance of auditory cortical responses (Figures 2B–2D and S2A; *p* < 0.001, *n* = 616). This provides a new lower bound on the proportion of the variance of A1 responses that can be captured by real-world models. Previously, analysis of response variability^30^ has suggested that the theoretical limit of A1 predictability is much higher than the actual prediction performance that models have been able to achieve. However, despite much effort and the use of sophisticated models,^18^^,^^31^ it has proven difficult to close this gap, raising the question of whether it is possible to approach the theoretical limits. PCMs show that it is possible to significantly close this gap and also quantify the balance between the linearity and nonlinearity of A1 neurons for the first time (Figures 2D, S2M, and S2N). Importantly, this also demonstrates that a major part of auditory cortical responses to complex sounds cannot be captured by STRFs (or by neural network models; Figure S4).

The high performance of the full PCM (i.e., predicting using IC+MGB+A1 units) could result from different cortical units having similar responses or because the responses of cortical neurons are well described by patterns of feedforward connectivity from subcortical neurons. We therefore asked to what degree a purely feedforward model (with inputs from IC and MGB) could account for this drastic improvement in our ability to predict A1 neural activity. We found that, as expected, the full model (Figure 2D) significantly outperformed the purely feedforward model (Figure 2E; *p* << 0.001, *n* = 616), demonstrating spectrotemporal nonlinearity introduced by A1 itself. Surprisingly, however, the difference in performance was fairly small, indicating that most of the superiority of the full PCM over the STRF can be achieved using only subcortical feedforward inputs to the auditory cortex (Figure 2E). This demonstrates that a large proportion of the nonlinearity in cortical responses (i.e., the variance unaccounted for by STRF models) actually arises because of nonlinear feedforward transformations in the subcortical ascending auditory pathway.

### Cortical responses arise from successive nonlinear transformations

**Figure 3:**
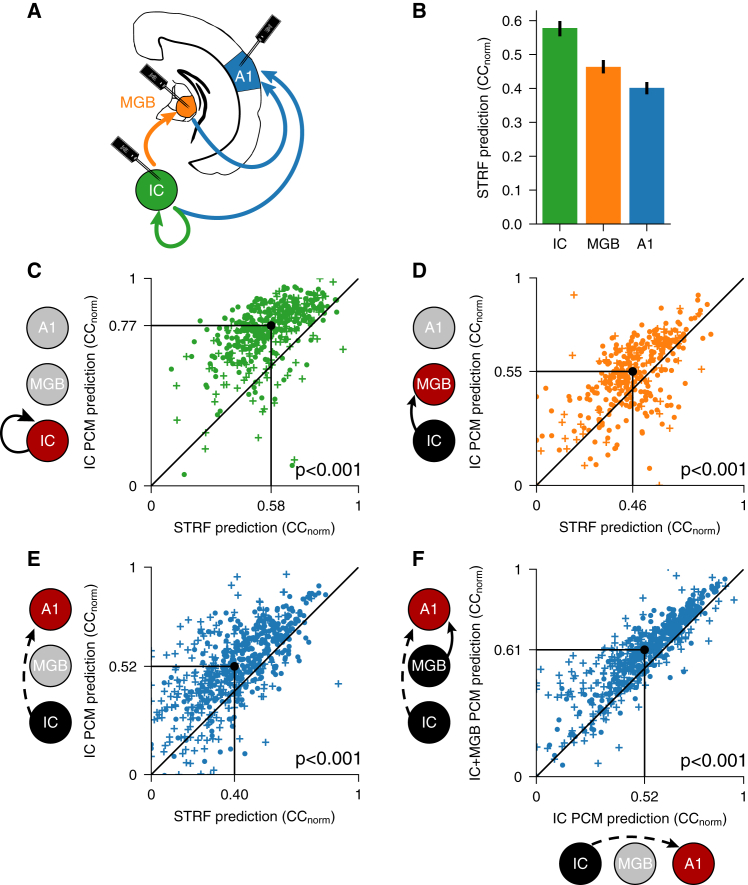
Representations of sound are nonlinearly transformed at each stage of the auditory pathway (A) Schematic of recording sites in the IC, MGB, and A1. Arrows indicate processing levels between which population communication models were applied. (B) Decreasing capacity of STRF models to predict neuronal responses across the ascending auditory pathway. Error bars are 95% nonparametric confidence intervals. (C) Comparison of prediction accuracy of IC responses between the population communication model (using IC units as the source population) and STRF models of the same units. Black dot indicates medians. (D) As in (C), but predicting MGB unit activity (using IC as the source population). (E) As in (C), but predicting A1 unit activity (using IC as the source population). (F) Prediction accuracy of A1 responses for the population communication model using both IC and MGB units as the source population (feedforward model) and the population communication model using IC units only as the source population. Plusses denote units recorded under anesthesia, and filled circles denote units recorded in awake animals. See also Figure S3.

We then set out to investigate where these nonlinear transformations occur along the subcortical ascending auditory pathway and how information is passed on to higher levels of auditory processing. As expected, we found that the predictability of auditory responses to complex sounds by STRFs decreased with every step along the auditory pathway (Figures 3A, 3B, and S2A–S2L; *p* < 0.001, *n*_IC_ = 432, *n*_MGB_ = 355, *n*_A1_ = 616), suggesting a progressive decrease in the linearity of spectrotemporal representation. This is not surprising because the IC is generally considered to be a processing stage with a more faithful representation of the spectrotemporal content than A1.^32^^,^^33^ What is unexpected, however, is that A1 responses are better predicted by the full PCM than IC responses are by the STRF model, suggesting that A1 responses have a much more faithful dependence on sound than previously appreciated, but this reliability is obscured by nonlinearities that spectrotemporal models cannot capture (Figures 2C, 3B, S2M, S2N, and S3).

Strikingly, we also found that predictions of IC unit activity by local IC population communication markedly outperformed STRF predictions of IC units (Figure 3C; *p* < 0.001, *n* = 432), indicating that a major nonlinear transformation of spectrotemporal information has already taken place by the time that auditory signals are represented at the level of the midbrain. These midbrain spectrotemporal representations are then passed on via the thalamus to cortical neurons through population communication, as demonstrated by the capacity of population communication from midbrain units to significantly improve predictions of both thalamic (Figure 3D; *p* < 0.001, *n* = 355) and cortical (Figure 3E; *p* < 0.001, *n* = 616) responses compared with STRF models.

Importantly, adding MGB units to the IC-only model (i.e., the feedforward model) further increased our ability to predict auditory cortical responses (Figure 3F; *p* < 0.001, *n* = 616). This suggests that an additional nonlinear transformation of acoustic information takes place in the thalamus and that this, in turn, contributes to the representation of complex sounds in A1.

Together, these findings demonstrate that nonlinear transformations take place at multiple steps along the ascending auditory pathway. The results of these transformations are passed on from level to level through population communication between hierarchical levels of the auditory pathway, ultimately shaping cortical representations of spectrotemporal information.

### Activity beyond receptive-field-based models in auditory cortex

**Figure 4:**
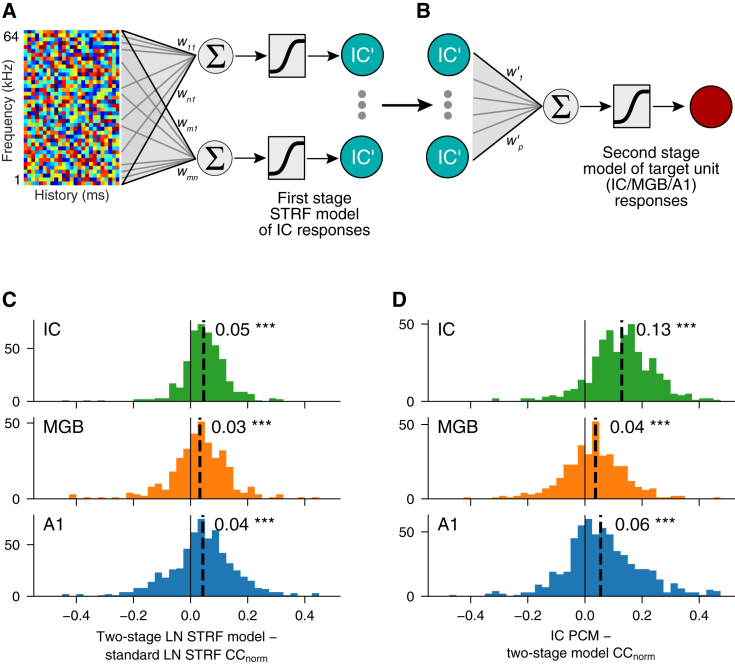
Two-stage models of ascending auditory connectivity (A and B) Schematic showing how two-stage biological neural network models are constructed. The first stage (A) consists of a population communication model of all IC units, using non-simultaneously recorded IC units as the source population. In the second stage (B), a population communication model is fitted that describes the response of each target neuron (in IC, MGB, or A1) in terms of the modeled responses (IC’) from the first stage. Taken together, the two stages describe the whole transformation from spectrogram to neural response. The structure is identical to a two-layer neural network model, but the hidden units are constrained to describe the responses of the recorded IC units. (C) Prediction performance of two-stage models for target neurons in IC, MGB, and A1, compared with STRF models. (D) Prediction performance of IC population communication model for target neurons in IC, MGB, and A1, compared with two-stage models. See also Figure S4.

To establish the extent to which PCMs incorporate new information about the responses of auditory neurons, we built two-stage biological neural network models (Figures 4A and 4B) of each neuron, where the first stage consisted of a set of linear-nonlinear STRF models of all IC neurons in our dataset and the second stage consisted of a PCM that used the modeled IC responses as input (instead of the real responses, as used in the previous version). This approach captures the degree to which PCM performance can be explained in terms of STRF models. We found that the two-stage models outperform standard linear-nonlinear STRF models (Figure 4C) but are substantially less predictive than normal PCMs (Figure 4D). This indicates that the PCMs embody neuronal characteristics—perhaps temporal or stimulus-specific adaptation—that are common to many neurons across the dataset but are not captured by linear-nonlinear STRF models, including in these hybrid STRF/PCM models.

The two-stage models have a similar structure to NRF models,^18^, but the outputs of the first stage are constrained to model the responses of real neurons rather than learning a representation. NRF models can capture nonlinear interactions between stimulus channels,^18^ but these are not well sampled by the spectrally random stimuli used here. As a result, NRF models do not consistently outperform linear-nonlinear STRF models on this dataset (Figure S4J). Two-stage models slightly outperform NRF models, indicating that constraining the hidden units by fitting them directly to IC responses is beneficial for predicting cortical responses (Figure S4L).

### PCMs are robust to experimental and analytical factors

To investigate the robustness of PCM performance, we varied numerous aspects of the models (Figure S5). We found that the pattern of performance is consistent across different datasets and subsets of data as well as different model parameters (nonlinearity, regularization, and time bin size). PCM performance saturates for source population sizes of around 100 units (Figure S5H), indicating that our sample size allows these models to approach maximum performance in all regions. We also found that there is no significant relationship between PCM performance and the firing rate or response reliability of the target neuron (Figures S4A–S4F). PCM performance is moderately dependent on the overlap between the tuning of the source population and the target neuron; however, the models can perform very well even when the spectral tuning overlap is low (Figures S4G–S4I). PCMs outperformed STRF models in both awake (Figure S5C) and anesthetized mice (Figure S5D). As the number of unique stimuli was increased, the performance advantage of PCMs over STRF models was maintained (Figure S5T), indicating that this advantage is not affected by dataset size. This demonstrates that PCMs are robust to a large range of experimental and analytical factors, including fitting parameters, number of cells recorded, size of stimulus space, and brain states.

### Cortical representations of sound are higher order and lossy

**Figure 5:**
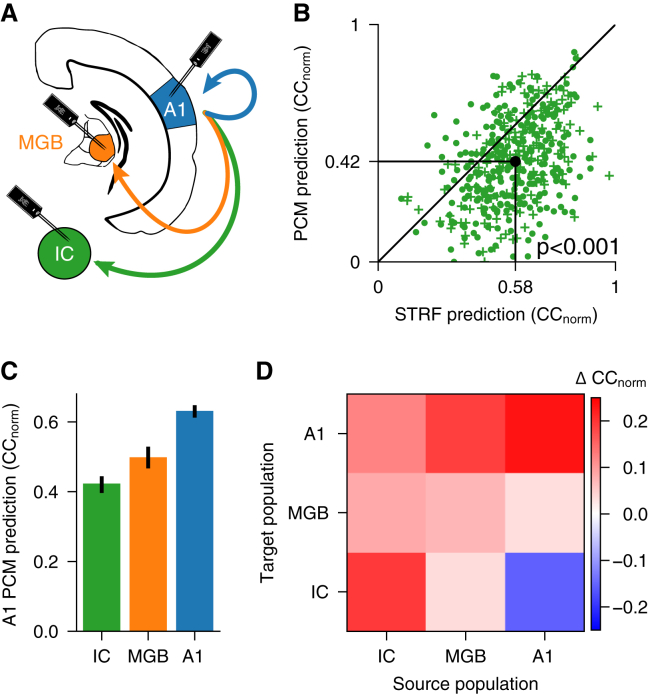
Nonlinear transformations along the ascending pathway are irreversible (A) Schematic of recording sites and descending connections modeled. (B) Comparison of prediction accuracy of IC unit responses for a population communication model in which A1 units were used as inputs versus STRF models of the same IC units. Black dot indicates medians. Plusses denote units recorded under anesthesia, and filled circles denote units recorded in awake animals. (C) Decreasing capacity of A1 units to predict neuronal responses at progressively earlier stages in the auditory pathway. Error bars are 95% nonparametric confidence intervals. (D) Difference (median) between prediction accuracy of population communication models and STRF models for units recorded in the IC, MGB, and A1 (target population) with source populations from different processing levels (see also Figures S2D–S2L). See also Figure S5.

We next assessed the role of descending population communication in shaping subcortical auditory spectrotemporal representations using models based on the responses of higher-level units as input for modeling lower-level units. We found that a model using cortical units as input (A1 PCM) poorly predicted both MGB and IC responses (Figures 5A–5C), with STRF models of IC responses substantially outperforming the predictions of the cortical PCM (Figure 5B; *p* < 0.001, *n* = 432). Indeed, subcortical representations could not be captured well by descending cortical population communication; the more ascending steps away the predicting population was, the worse the prediction became (Figures 5C and 5D; IC versus MGB, MGB versus A1, IC versus A1: all *p* << 0.001, *n*_IC_ = 432, *n*_MGB_ = 355, *n*_A1_ = 616), even when the longer latencies of cortical responses were taken into account (Figures S3D–S3F; see also Figure S5M). This suggests that the nonlinear transformations taking place along the ascending auditory pathway are irreversible: a higher-level (e.g., cortex) representation cannot be transformed into a lower-level (e.g., midbrain) representation. This implies that cortical representations are higher order, with information being discarded along the ascending auditory pathway.

### Subcortical responses depend on corticothalamic feedback

**Figure 6:**
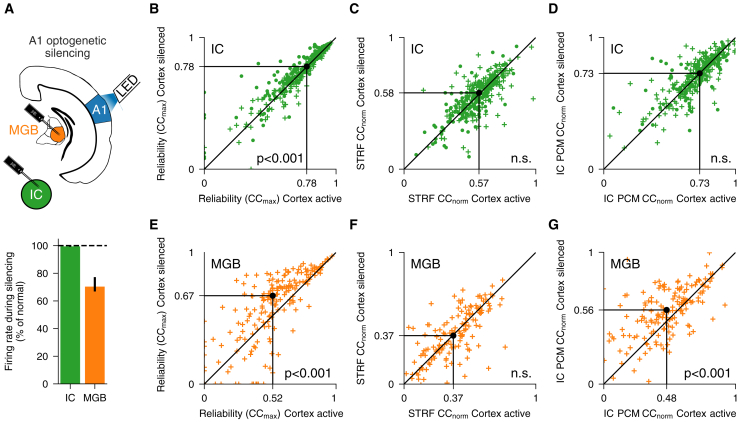
Corticofugal feedback selectively modulates nonlinear responses in MGB (A) Top: schematic of optogenetic silencing experiments. Bottom: cortical silencing decreases neuronal firing rate in IC and particularly MGB. Error bars are 95% nonparametric confidence intervals. (B) Trial-to-trial reliability (CC_max_) of IC units with and without auditory cortical silencing. Black dot indicates medians. (C) STRF prediction accuracy (CC_norm_) of IC units with and without auditory cortical silencing. (D) IC PCM prediction accuracy (CC_norm_) of IC units with and without auditory cortical silencing. (E–G) Same as in (B)–(D), but for MGB.

To better understand what information is sent back through the extensive descending corticofugal projections to thalamus and midbrain, we optogenetically silenced auditory cortex while recording the responses of an additional 749 units (19 mice) in the IC (*n* = 559) and MGB (*n* = 190). We tested the effects of cortical silencing on the population communication between the auditory midbrain and thalamus. Auditory cortical silencing significantly decreased the overall firing rate of MGB and IC neurons (although the effect in the IC was small; 0.6% decrease in IC, 29.7% decrease in MGB) (Figure 6A, lower). Although the recording probes first passed though the thin corticorecipient dorsal shell of the IC,^34^ most recordings were in the central nucleus, which may explain why cortical silencing had a much smaller effect on the midbrain. These findings therefore demonstrate that descending A1 connectivity primarily modulates the firing rate of the thalamic region from which it receives its ascending information. We also found that cortical silencing significantly increased the trial-to-trial reliability of units in both IC and MGB (although the effect in IC was again small) (Figures 6B and 6E), suggesting that cortex provides input to subcortical stations that varies from trial to trial and is independent of the spectrotemporal structure of the stimulus.

### Cortical modulation of nonlinear sound encoding in thalamus

STRF model prediction performance was not affected by auditory cortical silencing, after accounting for the change in trial-to-trial reliability using CC_norm_ (Figures 6C and 6F; *p* > 0.05, *n*_IC_ = 190, *n*_MGB_ = 559; STAR Methods). Crucially, however, the IC to MGB PCM performed significantly better after the cortex was silenced (Figure 6G; *p* < 0.001, *n*_IC_ = 190, *n*_MGB_ = 559), whereas the IC to IC model performance was unchanged (Figure 6D). This suggests that corticothalamic projections modulate nonlinear stimulus-dependent activity in MGB while leaving the linear representation of spectrotemporal information intact. Together, these findings reveal that the cortex can influence the activity of thalamic neurons in two distinct ways.

### Cortical control of functional coupling within subcortical areas

To further explore how descending corticofugal connections control the structure of subcortical activity, we constructed a generalized linear model (GLM) model of the moment-to-moment spiking activity from the stimulus inputs, both with and without including spike coupling filters between simultaneously recorded units^35^ (Figure S6A). We fitted this model to neural populations recorded in the midbrain and thalamus and investigated how functional spike coupling between subcortical units was affected by optogenetic silencing of cortex.

**Figure 7:**
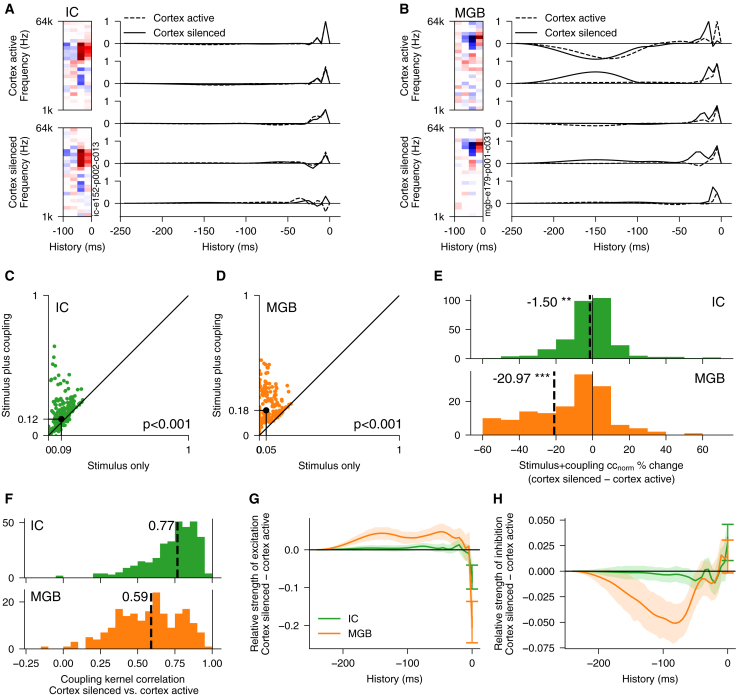
Cortex controls functional spike coupling between simultaneously recorded neurons in MGB and IC (A) Example IC unit STRF (left) and coupling filters of five simultaneously recorded IC units (right) with and without optogenetic cortical silencing. (B) Same as in (A), but for MGB. In each case, these filters were estimated from a single-trial spike coupling GLM model. (C) Comparison of stimulus-only model versus model that also incorporated coupling between simultaneously recorded IC units in predicting single-trial spike rates. Black dot indicates medians. (D) Same as in (C), but for MGB units. (E) Coupling of simultaneously recorded units adds more explanatory power for IC and especially MGB units when cortex is intact than when cortex is optogenetically silenced. (F) Coupling filters from simultaneously recorded units change significantly in IC and especially in MGB when cortex is optogenetically silenced. (G) Change in strength of excitatory components of coupling filters in IC and MGB over time after silencing auditory cortex. (H) Change in strength of inhibitory components of coupling filters in IC and MGB over time after silencing auditory cortex. Error bars are 95% confidence intervals. See also Figure S6.

We found that including coupling between units in this model increased the predictability of moment-to-moment spiking activity, implying that these responses depend not only on the stimulus (signal correlations) but also on the functional coupling between neurons (noise correlations) in thalamus and midbrain (Figures 7A–7D; with cortex active or silenced; for each condition, *p* << 0.001, *n*_IC_ = 190, *n*_MGB_ = 559). Silencing auditory cortex also significantly decreased the contribution of local functional coupling in explaining both thalamic and midbrain activity, with much stronger effects for the thalamus (Figure 7E; *p* < 0.001, *n*_IC_ = 190, *n*_MGB_ = 559; Figure S6). This suggests that the descending projections from cortex to thalamus and midbrain allow the orchestration of functional coupling between neurons within these circuits.

We also found that the structure of the functional coupling between neurons in IC and MGB was altered when cortex was silenced, with larger changes in MGB than in IC (Figure 7F; *p* < 0.001, *n*_IC_ = 190, *n*_MGB_ = 559). In particular, cortical inactivation decreased the fast (<10 ms) excitatory coupling between neurons in thalamus (and to a smaller but significant degree in the midbrain) but increased the slower (>10 ms) excitatory coupling (Figure 7G). Conversely, fast inhibitory coupling in thalamus was slightly decreased, whereas slower inhibitory coupling was strengthened when cortex was active (Figure 7H). This dynamic cortical control of functional coupling between neurons in the thalamus is consistent with a disruptive effect of silencing cortex on thalamocorticothalamic (excitatory) and thalamocortico-reticulothalamic (inhibitory) open loops between thalamic cells.

## Discussion

By modeling the responses of neurons in terms of their ascending inputs, we have shown that the representation of sound in the auditory cortex arises primarily from nonlinear transformations between the cochlea and the auditory midbrain, with the midbrain representation then being communicated to thalamic neurons, where further nonlinear processing takes place. These subcortical transformations, together with local cortical processing, produce a highly nonlinear representation of sound in the auditory cortex. Importantly, the transformations that occur along the ascending auditory pathway are irreversible, meaning that subcortical responses cannot be predicted from the cortical representation that they give rise to.

Although it is well known that spectrotemporal models fail to capture much of the stimulus-dependent response variance of auditory cortical neurons,^36^^,^^37^ it has proven difficult to improve these models in ways that capture this unexplained variance. NRF models modestly outperform simple STRF models for natural sound stimuli^18^ and perform similarly to STRF models for the complex spectrally random stimuli used here. This has led to the conclusion that much of the unexplained variance in cortical responses may be the result of cognitive factors. There has therefore been considerable focus in recent years on how behavioral task demands and brain state differences shape the sensitivity and spectrotemporal tuning properties of auditory cortical neurons.^8^^,^^27^^,^^38^^,^^39^^,^^40^

Here, we show that much of the unexplained variance reflects the failure of existing spectrotemporal models to adequately account for the nonlinear tuning properties of cortical neurons. PCMs are able to explain 70% more cortical response variance than spectrotemporal models, an improvement that is substantially larger than that achieved by attempts to incorporate nonlinearities into spectrotemporal models.^9^^,^^12^^,^^13^^,^^17^^,^^18^ This high performance is remarkably robust across variations of model and neural response parameters, multiple datasets, and subsets of the datasets, suggesting that it reflects neuronal behavior that is common across a large proportion of auditory neurons but is unexplained by existing models. A substantial portion of the previously unexplained variance is genuinely stimulus-dependent and predictable and must therefore reflect the structure of auditory stimuli. Because the additional variance cannot be accurately expressed by linear, linear-nonlinear, or network-based spectrotemporal models, it appears to reflect fundamentally higher-order representations of sound. Moreover, by demonstrating that we can predict the responses of A1 neurons to complex acoustic stimuli with considerably more accuracy than has hitherto been possible, our findings indicate that the auditory cortex does play a key role in faithfully representing sounds—albeit in a highly nonlinear way—rather than just the sensory and behavioral context in which those sounds occur.

By also building PCMs of descending connectivity, we have shown that A1 representations are higher order and cannot be converted back into subcortical representations by linear or neural network models. This indicates that the transformation of information from subcortical areas to cortex is lossy and irreversible. Surprisingly, although cortical neurons are known to follow rapid changes in auditory stimuli less well than subcortical neurons,^19^^,^^20^^,^^41^^,^^42^ these results are consistent even for time bins as long as 200 ms, suggesting that they cannot be accounted for by differences in temporal integration between auditory cortex and its thalamic inputs. This raises the possibility that rather than merely constructing a more complex representation of sound, the cortex also actively discards spectrotemporal information that is necessary for representation in subcortical areas but that may not be needed for higher-level processing. In particular, the information encoded in cortex is likely to be especially relevant for its well-known role in learning and other cognitive functions in which neuromodulatory inputs are implicated.^43^^,^^44^

Such transformations into higher-order representations are akin to what is found between thalamus and cortex in the visual system,^22^^,^^45^^,^^46^ but have been harder to identify in the auditory system. We find that these transformations occur between the midbrain and thalamus as well as between thalamus and cortex, and it seems likely that they also take place in the auditory brainstem. An important question for future research is whether comparable sequential transformations of information also take place in the other sensory systems. Applying the population communication framework introduced here to large-scale recordings from multiple levels of these systems could illuminate whether this is the case and enable the contribution of each processing stage to be quantified.

It is well known that the auditory cortex is the source of massive descending connections to auditory and other subcortical nuclei.^25^^,^^26^^,^^47^ Previous studies have shown that the activity and response properties of subcortical neurons can be altered by manipulating cortical activity.^48^^,^^49^^,^^50^^,^^51^^,^^52^^,^^53^ Nevertheless, our understanding of how descending corticofugal inputs contribute to auditory processing is incomplete. We found that silencing auditory cortex alters the moment-to-moment spike coupling between cells in the thalamus and midbrain, suggesting that descending corticofugal input may control the timing and balance of excitatory and inhibitory functional coupling in midbrain and thalamic circuits. This would allow for flexible orchestration of subcortical functional networks, potentially controlling which neuronal populations are synchronized and which are not, and, therefore, which stimulus representations are amplified and transmitted more effectively to cortex.^54^

Our optogenetic experiments also indicate that corticothalamic modulation selectively affects the higher-order (nonlinear) representations of sound in the thalamus, leaving linear spectrotemporal tuning intact. We observed much smaller effects of cortical inactivation on IC responses. This may be because our recordings were principally in the central nucleus rather than IC shell where appropriately timed ascending and descending inputs may be integrated nonlinearly.^55^ Such interactions between cortex and thalamus may happen through specific communication subspaces, as recently found between cortical areas in the visual system.^56^^,^^57^ The exclusive modulation of higher-order response properties suggests that feedback from the auditory cortex principally influences thalamic responses that are most closely related to its own higher-order sound representation. This is reminiscent of the so-called egocentric selection previously described in the auditory,^24^ somatosensory,^58^ and visual systems^59^ and may therefore be a general feature of corticofugal modulation in sensory systems and of feedback across brain areas in general.

Our results highlight two approaches that should lead to a greater understanding of information coding in auditory and other sensory systems. The first is to use the modeled responses of subcortical neurons to build better neural network models of neurons in the cortex. Network models are well suited to modeling nonlinear interactions between stimulus channels,^18^^,^^31^ but existing models do not capture the nonlinearities that are accounted for by PCMs. Given sufficiently rich datasets of natural stimuli, future hybrids of network and PCMs should be able to capture both stimulus interactions and neuron-neuron interactions, using biological constraints to identify where and how sensory information is transformed. The second is to investigate the contribution that the tuning properties of subcortical neurons make to the high performance of PCMs of information transmission between different processing levels. These approaches are likely to yield fundamental insights into the role of subcortical structures in shaping cortical responses, both in the auditory system and other sensory systems.

## STAR★Methods

### Key resources table

**Table:** 

REAGENT or RESOURCE	SOURCE	IDENTIFIER
**Bacterial and virus strains**		

AAV2/5-EF1a-DIO-hChR2(H134R)-eYFP	UNC vector core	N/A

**Deposited data**		

Data sets used in this paper	This paper	https://doi.org/10.6084/m9.figshare.25943725

**Experimental models: Organisms/strains**		

Mice – C57BL6/J	Envigo, Mice	N/A
Mice - GAD2-IRES-cre	Jackson Laboratories, USA	RRID: IMSR_JAX:010802
Mice - *VGAT-ChR2-YFP*	Jackson Laboratories, USA	RRID: IMSR_JAX:014548
Mice - *C57BL6/NTac.Cdh23*	MRC Harwell, UK	N/A

**Software and algorithms**		

Electrophysiological acquisition software	Custom MATLAB code	https://github.com/ben-willmore/benware
Python	N/A	N/A
KiloSort	https://github.com/MouseLand/Kilosort	N/A
glmnet-python	https://github.com/bbalasub1/glmnet_python/tree/master/glmnet_python	N/A
Population communication models, GLM, and STRF modeling code	This paper	https://github.com/ben-willmore/population-communication-models

**Other**		

Silicon probes	NeuroNexus Technologies Inc.	4 × 8, 8 × 8, or 2 × 32 configuration
Neurodigitizer and preamplifier	Tucker-Davis Technologies	PZ5
Data processor and real-time controller	Tucker-Davis Technologies	RZ2 BioAmp processor
Stimulus delivery controller	Tucker-Davis Technologies	RX6 Multifunction processor

### Resource availability

#### Lead contact

Further information and requests for code or data should be directed to Michael Lohse (m.lohse@ucl.ac.uk).

#### Materials availability

This study did not generate new unique reagents.

#### Data and code availability

• Neurophysiological data have been deposited to figshare and are publicly available as of the date of publication at doi: https://doi.org/10.6084/m9.figshare.25943725.
• Original code used to generate analyses and figures presented in this manuscript has been deposited on GitHub and is publicly available as of the date of publication at https://github.com/ben-willmore/population-communication-models.
• Any additional information required to reanalyze the data reported in this paper is available from the lead contact upon request.

### Experimental model and subject details

All animal experiments conformed to ethical standards approved by the Committee on Animal Care and Ethical Review at the University of Oxford and were licensed by the UK Home Office (Animal Scientific Procedures Act, 1986, amended in 2012).

#### Mice

A total of 47 mice were used in this study. Four strains of male and female mice were used: *C57BL6/J* (Envigo, UK), *GAD2-IRES-cre* (Jackson Laboratories, USA), *VGAT-ChR2-YFP* (Jackson Laboratories, USA), and *C57BL6/NTac.Cdh23*.^60^ *C57BL6/J*, *GAD2-IRES-cre*, and *VGAT-ChR2-YFP* were 7–12-weeks old at the time of data collection, and *C57BL6/Ntac.Cdh23* were 10–20-weeks old at the time of data collection. All experiments were carried out in a sound-attenuated chamber.

### Method details

#### Stimuli

Stimuli were presented with a Tucker-Davis Technologies (TDT) RX6 Multifunction processor at a sample rate of ∼200 kHz. Sounds were amplified by a TDT SA1 stereo amplifier and delivered via a modified Avisoft ultrasonic electrostatic loudspeaker (Vifa) positioned ∼1 mm from the ear canal. The sound presentation system was calibrated to a flat (±1 dB) frequency-level response between 500 and 64,000 Hz.

Stimuli consisted of spectrotemporally complex dynamic random chords (DRCs) with individual chords having a duration of 25 ms (including 5 ms on and off ramps) and comprising 25 superposed frequencies, logarithmically spaced between 1000 and 64,000 Hz (1/4 octave intervals). The tones of the DRC were played at sound levels that were randomly drawn from one of two uniform distributions: 30–50 dB sound pressure level (SPL) (low contrast) or 20–60 dB SPL (high contrast). The mean of the distribution was therefore constant, at 40 dB SPL. The logarithmic statistics of the decibel scale have been found to better match the statistics of natural sounds.^21^^,^^61^ The overall sound level of the DRCs was calibrated to be 79–83 dB SPL. The total number of chords was 6,400 (dataset H in Figure S5; repeated 8 times, spread across multiple 40 s trials) or 6,000 (dataset O in Figure S5; repeated 10 times, spread across multiple 5 s trials in optogenetic experiments), with inter-trial intervals of 2–10 s.

#### In vivo extracellular recordings

We carried out extracellular recordings using 32- or 64-channel silicon probes (NeuroNexus Technologies Inc.), in a 4 × 8, 8 × 8, or 2 × 32 electrode configuration. Electrophysiological data were acquired on a Tucker-Davis technologies (TDT) RZ2 BioAmp processor and collected and saved using custom-written Matlab code (https://github.com/ben-willmore/benware). Only a single brain area (IC, MGB or AC) was recorded in any session. The numbers of units were as follows:

**Table:** 

Dataset H	Units	Units with noise ratio ≤200	Awake penetrations	Anesthetized penetrations	Units per penetration		
					Minimum	Median	Maximum
IC	448	440	8	5	23	33	54
MGB	418	362	7	5	5	29	81
AC	695	639	11	10	12	33	57

**Table:** 

Dataset O	Units	Units with noise ratio ≤200	Awake penetrations	Anesthetized penetrations	Units per penetration		
					Minimum	Median	Maximum
IC	576	559	6	10	19	35	52
MGB	217	190	0	5	34	44	55

##### Awake recordings

For awake recordings, we chronically implanted a recording chamber under isoflurane (1.5–2% in oxygen) general anesthesia together with administration of meloxicam (5 mg/kg) and dexamethasone (Dexadreson, 2-3mg/kg). The recording chamber consisted of a metal cylinder positioned over a craniotomy, with a lightly attached circular window in order to close the recording chamber. We placed the recording chamber around craniotomies over IC (centered ∼5 mm posterior from bregma and ∼1 mm lateral from midline), A1 (centered ∼2.5 mm posterior from bregma and ∼4.5 mm lateral from midline), or the cortex above MGB (centered ∼3 mm posterior from bregma and ∼2.1 mm lateral from midline), together with a head bar and a reference (silver wire) in the contralateral hemisphere. We then fixed the implant to the skull using dental adhesive resin cement (Super Bond C&B). Following full recovery, on a subsequent day the mouse was head-fixed, the recording chamber was opened, and the exposed dura mater was kept moist with saline. A sterile recording probe was then acutely inserted into the recording site of interest. All recordings were performed in the right hemisphere.

In the mouse, the dorsal surface of the IC is not covered by the cortex and is very distinct. The craniotomies over the IC were always large enough to see the entire exposed IC surface, so we could visually target the probes. We inserted probes into the IC, and then advanced them until all sites were judged to be in the central nucleus of the IC (CNIC). We confirmed this by checking for the clear dorsoventral tonotopic gradient in the STRFs that is indicative of this nucleus. The overlying dorsal shell of IC is relatively thin in this region,^34^ but a small proportion of recording sites may have been located here. We also estimated frequency response areas using tones, which confirmed the presence of dorsoventral tonotopic gradients with narrow tuning (data not shown). When we were positioning the electrode array, we observed tightly-locked multiunit responses to noise stimuli, characteristic of CNIC neurons, and post-mortem inspection of the midbrain confirmed that the probe had indeed been located in the CNIC.

Prior to insertion into auditory thalamus, the probe was coated in DiI (Sigma-Aldrich) for subsequent histological verification of the recording site. Recording sites were confirmed as being located in auditory thalamus if multiunit activity responded to broadband noise and was frequency tuned when the tip of the probe was ∼2.5–3.5 mm below the brain surface. Auditory thalamic recordings were subsequently attributed to the lemniscal ventral subdivision of the MGB (MGBv) by histological investigation of recording sites. Based on an immunohistochemical study by Lu et al.^62^ on the shape and size of subdivisions of the mouse auditory thalamus, we allocated recording sites to the MGBv if they responded reliably to DRC stimulation on electrode channels < 500 μm from the lateral border of the MGB.

Finally, A1 was identified by robust neuronal responses to broadband noise bursts, and a caudo-rostral tonotopic axis.

##### Anesthetized recordings

For experiments carried out under anesthesia, mice were anesthetized with an intraperitoneal injection of ketamine (100 mg kg^−1^) and medetomidine (0.14 mg kg^−1^). We also administered intraperitoneal injections of atropine (Atrocare, 1 mg kg^−1^) to prevent bradycardia and reduce bronchial secretions, and dexamethasone (4 mg kg^−1^) to prevent brain edema. Prior to initial surgery, bupivacain was administered as an analgesic under the scalp. The depth of anesthesia was monitored via the pedal reflex and small additional doses of the ketamine/medetomidine mix were given subcutaneously approximately every 15 min once the recordings started (∼1–1.5 h post induction of anesthesia). The dosage of individual top-ups depended on the depth of anesthesia at the time, but corresponded to ∼50 mg kg^−1^ h^−1^ of ketamine and ∼0.07 mg kg^−1^ h^−1^ of medetomidine. All recordings were performed in the right hemisphere. A silver reference wire was positioned in the visual cortex of the contralateral hemisphere, and a grounding wire was attached under the skin on the neck. The head was fixed in position with a metal bar acutely attached with bone cement to the skull over the left hemisphere. We then made 2-mm diameter circular craniotomies above the IC (centered ∼5 mm posterior from bregma and ∼1 mm lateral from midline), over the visual cortex for auditory thalamic recordings (centered ∼3 mm posterior from bregma and ∼2.1 mm lateral from midline), and/or over the auditory cortex (centered ∼2.5 mm posterior from bregma and ∼4.5 mm lateral from midline). Following exposure of the brain, the exposed dura mater was kept moist with saline. The silicon probe was then inserted carefully into the recording site of interest, and allocated similarly to the description in the awake recordings section.

#### Optogenetic silencing of auditory cortex

The data for the optogenetic silencing experiments were also used in Lohse et al.,^14^ where results confirming electrophysiologically effective silencing of activity are reported.

To transiently silence the activity of auditory cortical excitatory neurons, we employed either a transgenic or a viral approach to express ChR2 in auditory cortical inhibitory neurons. *VGAT-ChR2-YFP* mice express ChR2-YFP in GABAergic neurons throughout the adult brain. Optogenetic activation of cortical inhibitory neurons is the most effective available method for inhibiting cortical activity at sub-second time resolution over the time window required for this study and has been used extensively to transiently silence excitatory activity (including corticofugal outputs) in cortical areas in mice. Viral injection surgeries were performed under isoflurane (∼1.5%) anesthesia, with the animal positioned in a stereotaxic frame (Kopf instruments, USA). For viral transfection, we injected *AAV5-DIO-ChR2-eYFP* (UNC gene therapy vector core) into the auditory cortex of *GAD2-IRES-cre* mice. We injected ∼400 nl of virus, spread over three locations (spaced caudal-rostrally ∼400 μm apart) at three depths (700, 500, and 300 μm from cortical surface), to ensure widespread expression in auditory cortex. Mice were used for electrophysiological recordings > 4 weeks post injection of virus. This ensured strong expression of ChR2-eYFP in the auditory cortex.

For optogenetic silencing, we exposed the auditory cortex to blue (470 nm) LED light. This was achieved by placement of a 200 μm (*VGAT-ChR2-YFP* experiments) or 1 mm optical fiber (*GAD2-cre* + viral ChR2 experiments) immediately above the dura mater over the auditory cortex to allow for blue light exposure to ChR2-expressing cells. For silencing of auditory cortical activity during recordings in MGBv or CNIC, we stimulated with blue light at 40 Hz frequency using sinusoidal waves or 15 ms pulses (10 ms gaps). When recording from auditory cortex, we stimulated with blue light at 40 Hz using either sinusoidal waves or 15 ms pulses (10 ms gaps) or constant light stimulation. Light power was ∼5–7 mW mm^−2^ at the tip of the fiber. We found that light stimulation (40 Hz (sinusoid or pulsed) or constant light) effectively silenced activity in auditory cortical neurons by driving inhibitory neurons for the duration of the DRC stimulation (5 s) (see Lohse et al.^14^).

#### Histology

For post-mortem verification of the electrophysiological recording sites and viral expression patterns, mice were overdosed with pentobarbital (100 mg/kg body weight, i.p.; pentobarbitone sodium; Merial Animal Health Ltd, Harlow, UK) and perfused transcardially, first with 0.1 M phosphate-buffered saline (PBS, pH 7.4) and then with fresh 4% paraformaldehyde (PFA, weight/volume) in PBS.

#### Spike sorting

We clustered potential neuronal spikes using KiloSort (https://github.com/cortex-lab/KiloSort). Following this automatic clustering step, we manually inspected the clusters in Phy (https://github.com/kwikteam/phy), and removed noise (movement artefacts, optogenetic light artefacts etc.). We assessed clusters according to suggested guidelines published by Stephen Lenzi and Nick Steinmetz (https://phy-contrib.readthedocs.io/en/latest/template-gui/#user-guide).

### Quantification and statistical analysis

#### Modeling

After spike sorting, data collation was conducted in MATLAB, after which data was analyzed using Python code based on NumPy, scikit-learn and glmnet-python (https://github.com/bbalasub1/glmnet_python/tree/master/glmnet_python).

##### Neural responses

For each unit, we counted spikes in 25 ms time bins for the STRF and population communication models (corresponding to the chord length of the DRC stimuli), giving matrix *y*_*dt*_. For the single-trial Poisson Generalized Linear Model, spikes were counted in 5ms bins. Where appropriate, we averaged these counts over all trials to compute the peristimulus time histogram (PSTH), resulting in vector *y*_*t*_.

##### Unit selection criteria

We aimed to include as many units as possible in our analyses. For GLM spike coupling models (Figure 7), we included all units. For STRF and population communication models relying on trial-averaged responses, we excluded only units where responses were very unreliable (possibly corresponding to poorly isolated clusters, or simply not reliably encoding auditory information across trials). To achieve this, we measured reliability using the noise ratio^10^^,^^63^ (NR) of the responses, *y*_*dt*_, of each unit. Each unit was included in STRF, NRF and PCM analyses if it had a noise ratio of < 200 across the entire dataset (1441 out of 1561 units in dataset H). In order to make unbiased comparisons between different models, we also excluded from all analyses in Figures 1, 2, 3, 4, and 5 any unit where any model failed to produce a fit. This meant that a further 38 units were excluded from these analyses, giving a total of 1403 included units. For the optogenetic STRF and PCM analyses in Figure 6, units with noise ratio < 200 were included (749 out of 793 units from dataset O). For the GLM analyses (Figure 7), where single-trial variability should not be excluded, we included all 793 units from dataset O.

##### Testing on a held-out data set

To fit and test the STRF and population communication models, we divided the stimulus into *n* equally sized contiguous segments (*n*=16 for Figures 1, 2, 3, 4, and 5, *n*=15 for Figures 6 and 7). For every fit, models were trained on neuronal responses to the first (*n*-1) segments, and hyperparameter selection was carried out on a subset of the first (*n*-1) segments. Models were tested on the remaining (*n*^th^) segment, so all correlation coefficient values are for prediction on a “held-out” dataset, i.e., a subset that had not been used in any part of the training procedure. This approach means that the reported correlation coefficients are resistant to overfitting – i.e., models with greater numbers of regressors do not have a built-in advantage over models with fewer regressors. Due to the computation time required, we did not fit every model on all *n* folds of the data, though our main results were validated on all folds.

Our stimuli (dynamic random chords) had uncorrelated spectrograms at the time resolution of our analysis, and so, for all models, there are no stimulus correlations that might interfere with our model estimation. Also, since the stimuli were frozen, there were no differences between conditions. However, we did not have any control over the correlation structure of the neuronal responses, and because different neurons respond in correlated ways to structure in the stimuli, neuron-neuron correlations would be expected to arise. Thus, when the neuronal responses are used as regressors in PCMs, neuron-neuron correlations become relevant. The use of regularized regression to fit the models reduces the effects of these correlations. Importantly, since we used a held-out test set to evaluate our models, any artifacts resulting from these correlations would be expected to decrease performance on the test set (since the artifactual structure would not generalize to new data). This analysis is therefore unlikely to exaggerate the performance of population communication models relative to STRF models.

For GLM fitting, where trial-to-trial variability is included, models were trained on 5 concatenated single-trial responses to the entire stimulus. A held-out test set of 2 concatenated single-trial responses to the entire stimulus was used for model evaluation.

##### Model evaluation

The prediction performance of STRF and population communication models was evaluated on the held-out test set using normalized correlation coefficient^30^ (CC_norm_), i.e., the correlation coefficient between the predicted and actual responses after estimated trial-to-trial variability has been excluded. For optogenetic STRF and PCM comparisons (Figures 6C–6G), the normalization factor (CC_max_) was calculated separately for the cortex active and cortex silenced conditions, so that CC_norm_ values take into account variation in neuronal reliability between the two conditions. For GLM models, trial-to-trial variability is of interest and cannot be excluded. For these models, prediction performance was evaluated on the held-out test set using the standard Pearson correlation coefficient.

##### Linear-nonlinear STRF models

We characterized the power in each stimulus using the raw sound level (dB SPL) vs time values from which the DRCs were originally created. These values constitute a log-spectrogram (referred to as a cochleagram), *X*_*tf*_, where *t* indexes time in 25 ms steps, and *f* indexes the stimulus frequencies. The spectrogram contains complete information about the time-varying frequency content of the stimulus. We ‘tensorized’ the stimulus matrix by adding an extra dimension containing the unrolled stimulus history (over the most recent 13 time steps) at each time point, resulting in a spectrogram tensor, *X*_*tfh*_, where *h* indexes stimulus history. We then estimated a linear model consisting of a spectrotemporal kernel, *k*_*fh*_, and offset, *a*_0_, which is applied to the spectrogram tensor to produce a linear estimate, *z*_*t*_, of the time-varying neuronal responses:

$$z_{t}=a_{0}+\sum_{f,h}X_{tfh}\cdot k_{fh}$$

The values of *k*_*fh*_ and *a* were optimized using scikit-learn to minimize the mean square error between *z*_*t*_ and *y*_*t*_, subject to L2 regularization. Regularization strength was determined by a hyperparameter, *λ*, whose value was selected by cross-validation on a held-out subset of the training data (not overlapping with the test set). Finally, we applied a logistic output nonlinearity, which transforms the linear model output, giving the final modeled neuronal responses, ${\hat{y}}_{t}$:

$${\hat{y}}_{t}=a+\frac{b}{1+\exp\left(-(z_{t}-c\right)/d)}$$

Parameters *a*, *b*, *c* and *d* were optimized by gradient descent to minimize mean-square error between ${\hat{y}}_{t}$ and *y*_*t*_.

##### Linear-nonlinear population communication models

The linear-nonlinear population communication models were identical in form and optimization to the linear-nonlinear STRF models and differed only in the inputs to the regression procedure. Thus, these models consisted of a linear component which described the mean PSTH, *y*_*t*_, in terms of the tensorized PSTHs of the input neurons, *X*_*tuh*_, where *u* indexes neuron number. This was followed by a logistic output nonlinearity, as above.

The response history of input neurons was tensorized over 8 history steps, as well as 5 future steps. Neurons at increasing levels of the auditory system tend to have increasing response latencies. If the future time steps had not been included, PCMs using long-latency source populations would be expected to fail to predict short-latency responses, merely because the appropriate latencies were not present in the models. We wanted to be sure that the poor performance of descending PCMs was not due to this. We therefore included enough future latencies to allow for expected differences in neuronal latency. We also included a control model with no future latencies as Figure S5Q.

The source populations for the PCMs were taken only from non-simultaneously recorded neurons. If simultaneously recorded data are included (Figure S5M), PCM performance increases. This improvement is likely to be largely due to noise correlations, reflecting shared variability between simultaneously recorded neurons. However, we cannot rule out the possibility that experimental factors (shared non-neural variability) may inflate the PCM performance for simultaneously recorded data.

##### Analysis of STRF models

*Tuning width*: We summed the positive coefficients of each STRF over time to get a frequency tuning curve, and counted the number of values in this curve that were greater than 0.25x the maximum (Figure S1E).

*Best frequency*: We summed the positive coefficients of each STRF over time to get a frequency tuning curve. We interpolated this curve by a factor of 10 using a cubic spline, and found the frequency that corresponded to the peak (Figures S1F–S1I).

*Summed receptive field overlap*: We summed the positive coefficients of each STRF over time to get a frequency tuning curve. We used the dot product to calculate the overlap between the target neuron’s tuning curve and that of each member of the source population. The summed receptive field overlap for each target neuron (Figures S4J–S4L) was the sum of these values.

##### Network Receptive Field models

For Network Receptive Field (NRF) models,^18^ stimuli and neuronal responses were processed exactly as for the linear-nonlinear models. The network model (built using PyTorch) contained a single hidden layer of 2, 4, 8 or 16 hidden units with logistic activation functions, and an output unit with logistic activation function. The number of hidden units was treated as a hyperparameter which was chosen based on prediction performance (mean-square error) on a held-out subset of the training data (not overlapping with the test set).

##### Single-Trial Poisson Generalized Linear Model with Spike Coupling

The single-trial Poisson Generalized Linear Model (GLM) analyses used a Poisson GLM with dependence on stimulus and spike history, as well as coupling between neurons.^35^^,^^64^ To capture stimulus dependence, the design matrix contained the sound level history of the most recent 5 chords (125ms) at 5ms resolution. To capture coupling between neurons, we convolved the responses of each simultaneously recorded neuron with a set of 12 basis functions spanning 250ms (Figure S6B; delta functions at latencies 5ms and 10ms, followed by logarithmically-spaced raised cosine filters; peak latency of longest filter = 150ms). Finally, to capture spike history, we convolved the spike history of the neuron with a set of 7 basis functions spanning 100ms (Figure S6C; delta functions at latencies 5ms and 10ms, followed by logarithmically-spaced raised cosine filters; peak latency of longest filter = 50ms). GLMs were fitted using glmnet-python. The fitted coupling and spike history filters were reconstructed by multiplying each filter by the corresponding fitted coefficient, resulting in a time-varying filter relating the target neuron’s response to the recent history of each regressor units’ spiking activity.

Unlike the PCM models, the GLM models are specifically intended to capture noise correlations, i.e., shared variability between the responses of simultaneously recorded neurons on single trials. We therefore only included simultaneously recorded neurons as inputs to the GLM models. Since only one brain area was recorded at a time, this restricted us to investigating within-area spike coupling effects.

#### Statistical inference

Unless specified otherwise, all *p-value*s were estimated using non-parametric Wilcoxon signed-rank tests (paired samples) or Mann-Whitney U tests (independent samples). Ninety-five percent non-parametric error bars were estimated using sampling with replacement to obtain a distribution of bootstrapped median values, and from this distribution the 2.5 and 97.5 percentiles were identified to create 95% confidence intervals around the median.

## Supplemental information

Document S1. Figures S1–S6

Document S2. Article plus supplemental information
