# Supplementary material for: Subcortical origin of nonlinear sound encoding in auditory cortex
Source: Curr Biol. 2024 Aug 5;34(15):3405–3415.e5. doi: 10.1016/j.cub.2024.06.057 (PMC11512679; doi:10.1016/j.cub.2024.06.057)
Supplement: Document S1. Figures S1–S6 [file mmc1.pdf]

**Current Biology, Volume *34***

## Supplemental Information

# Subcortical origin of nonlinear sound encoding in auditory cortex

**Michael Lohse, Andrew J. King, and Ben D.B. Willmore**

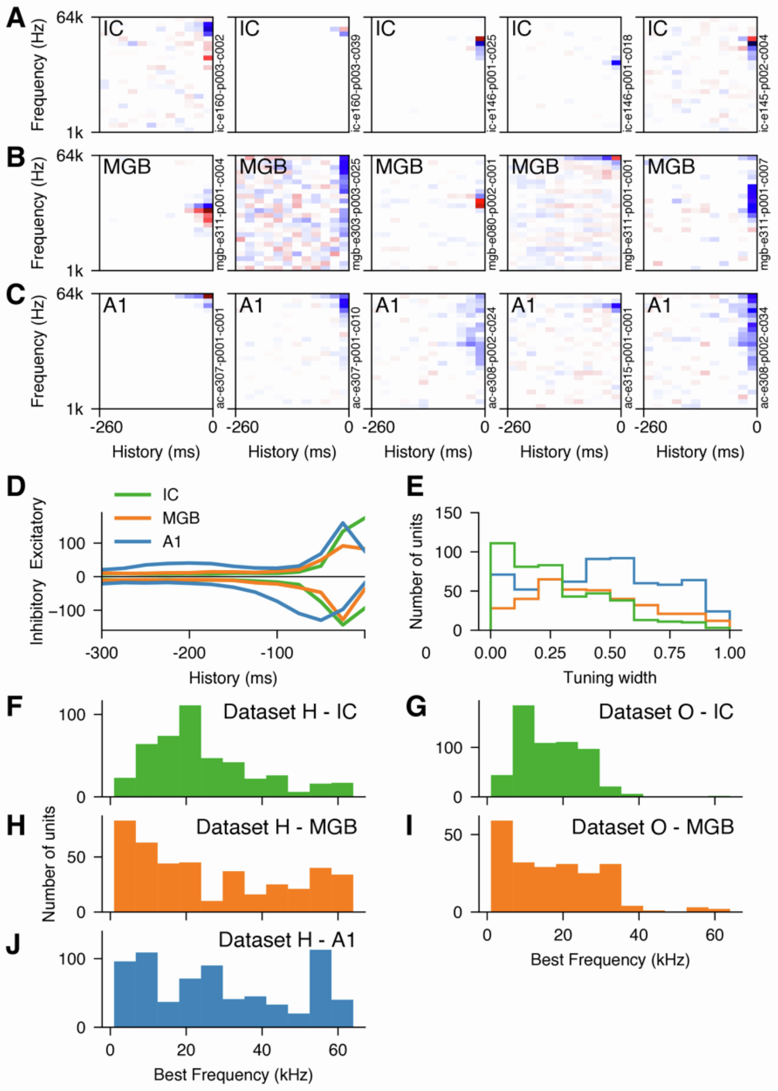


**Figure S1: STRF model characteristics over the auditory hierarchy, Related to Figure 1.**
(A-C) Examples of STRFs in IC, MGB and A1, respectively.
(D) Time course of excitatory (top) and inhibitory (bottom) coefficients, summed across all STRFs for each brain area, showing the increase in response latency at higher levels of the auditory hierarchy.
(E) Tuning width of STRFs (see Methods), showing an increase across the hierarchy.
(F-J) Distributions of best frequencies (see Methods) of units in IC, MGB and A1 for datasets H and O.

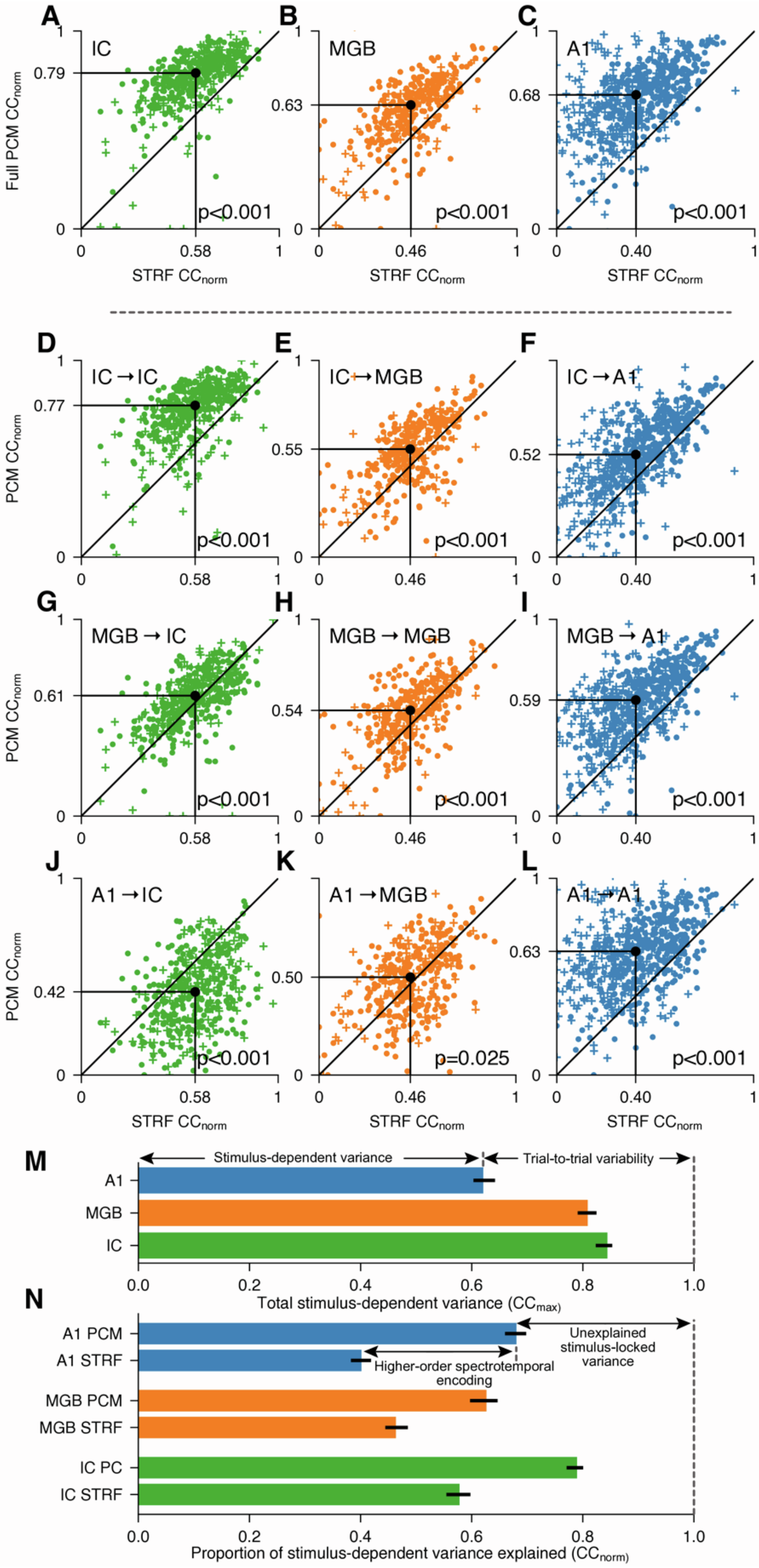


**Figure S2: Additional variance explained by the population communication models compared to STRF models, Related to Figure 2.**
(A-C) Comparison of prediction performance of full population communication model (taking inputs from non-simultaneously-recorded units in IC, MGB and A1; y-axis), relative to STRF model (x-axis), for predicting responses in IC, MGB and A1, respectively.
(D-L) Comparison of prediction performance of single-area population communication models (y-axis), relative to STRF model (x-axis). The source population and target areas are shown in the top-left of each plot (source population → target).

(M) Total stimulus-dependent variance ($CC_{max}$) in IC, MGB and A1, respectively. The difference from 1 is a measure of how much variance in each area is not stimulus-dependent, i.e., trial-to-trial variability in neural responses.
(N) Median proportion, $CC_{norm}$, of the stimulus-dependent variance ($CC_{max}$) that can be explained by STRF and population communication models for each brain region. The performance of the STRF model is a measure of how much variance can be explained by simple spectrotemporal models. The additional performance of the population communication model indicates the proportion of neuronal responses that can be explained by higher-order spectrotemporal encoding, which is stimulus-driven but cannot be modeled with simple spectrotemporal models. The remaining variance is stimulus-dependent, but remains unexplained.

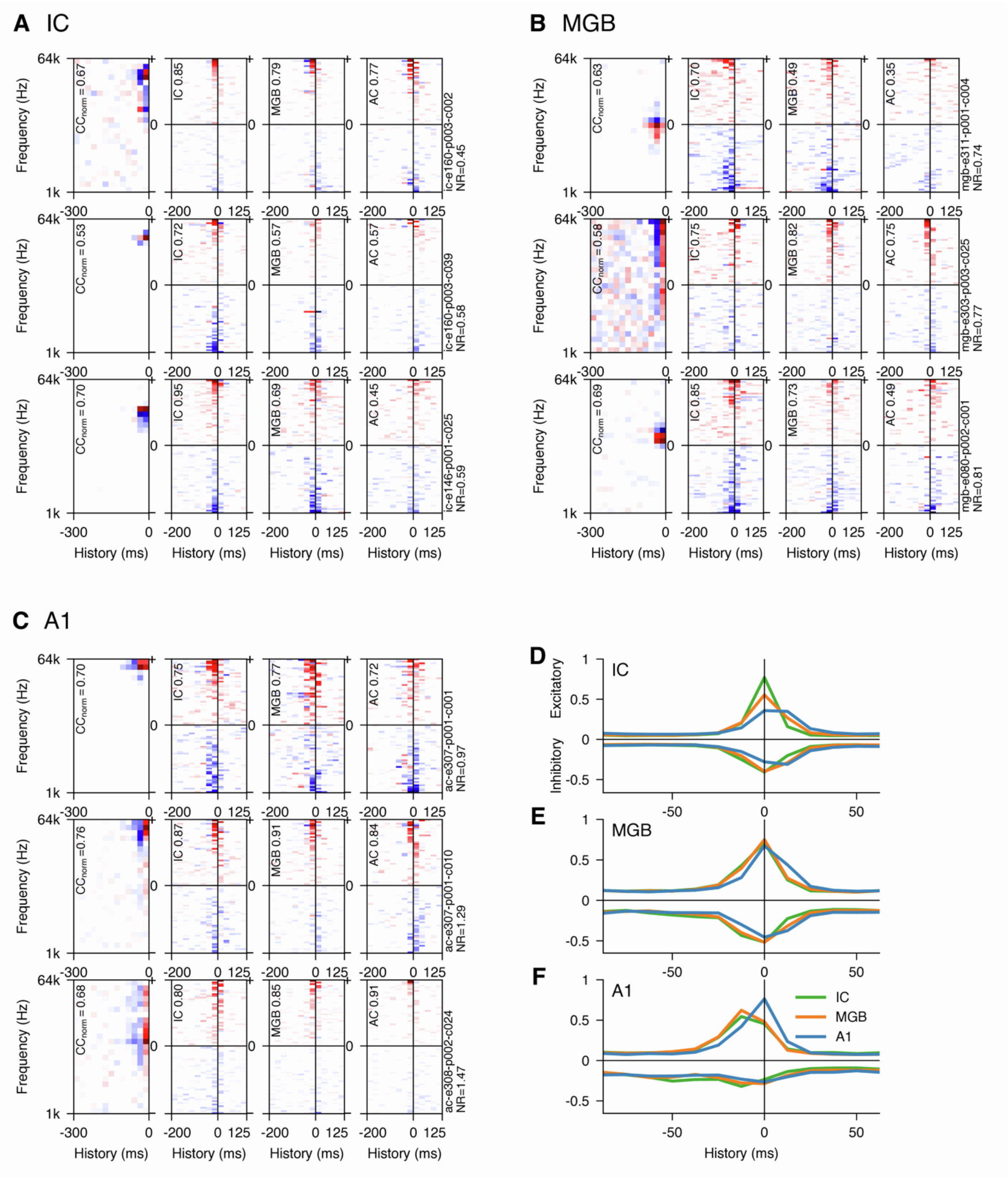


**Figure S3: Structure of STRF and population communication model kernels across the auditory hierarchy, Related to Figure 3.**

(A) Examples of STRFs and population communication model kernels for three example IC neurons. The left column shows the STRF model kernel, and the subsequent three columns show population communication model kernels using IC, MGB and A1 units as the source population, respectively. $CC_{norm}$ values for each model are shown in the top left. For population communication model kernels, only the source units with the 20 highest (above the axis) and 20 lowest (below the axis) summed coefficient values are shown, in descending order of summed coefficient value.

(B, C) Similar examples for MGB and A1 units, respectively.

(D) Time course of excitatory (above x-axis) and inhibitory (below x-axis) coefficient values in population communication models of IC neurons, summed over the source population in single-area population communication models. The mode offset for IC-to-IC coefficient values is 0, and increases

for source units in higher auditory areas (MGB and A1). This is expected, given the increasing latency of typical neural responses across the auditory pathway.
(E, F) Similar plots for MGB and A1 target units, respectively.

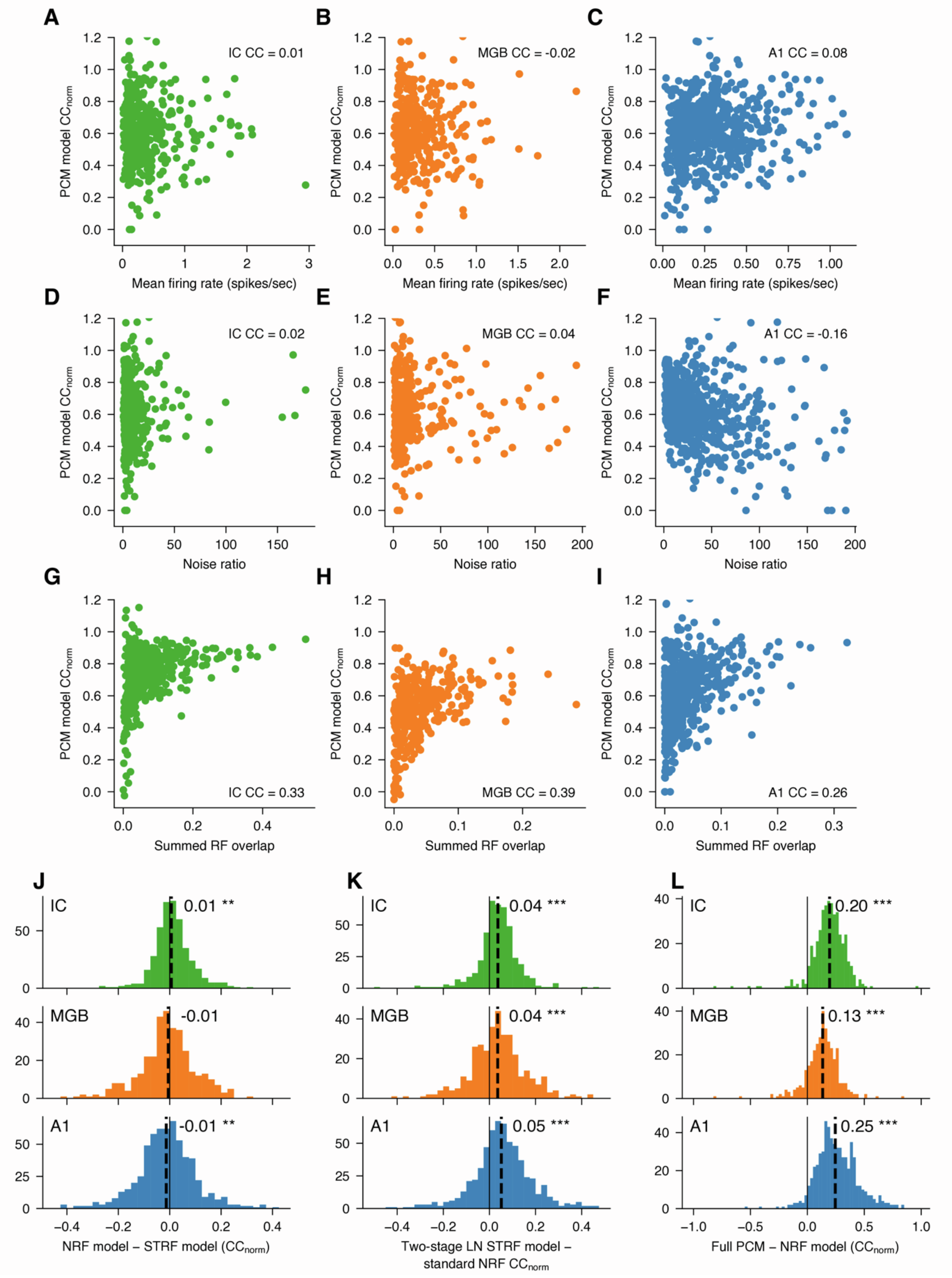


**Figure S4: PCM and NRF model performance, Related to Figure 4.**

(A-C) PCM performance against mean firing rate of the target neuron for IC, MGB and A1.

(D-F) PCM performance against noise ratio of the target neuron for IC, MGB and A1.

(G-I) PCM performance against the summed receptive field overlap between the target neuron and the source population (see Methods).

(J) Relative performance ($CC_{norm}$) of Network Receptive Field (NRF) models and linear-nonlinear STRF models in predicting responses of neurons in IC, MGB, A1, respectively. In this study, we used

linear-nonlinear STRF models as our baseline for evaluating the performance of population communication models. It is conceivable, therefore, that the apparently high predictive power of population communication models merely reflects poor performance of our STRF models. To ensure this is not the case, we also fitted NRF models, which describe the responses of each neuron using a neural network whose input is the time-varying cochleagram (log-spectrogram). We find that, for this dataset (where the stimuli are spectrally random), the NRF model outperforms the STRF model for some neurons, and underperforms for others, but there are only minor differences in predictive power across the neural population in all three areas.

(K) Relative performance of two-stage models and NRF models in predicting responses of IC, MGB, A1, respectively.

(L) Relative performance of Full (IC, MGB and A1 source populations) PCM models and NRF models in predicting responses of IC, MGB, A1, respectively.

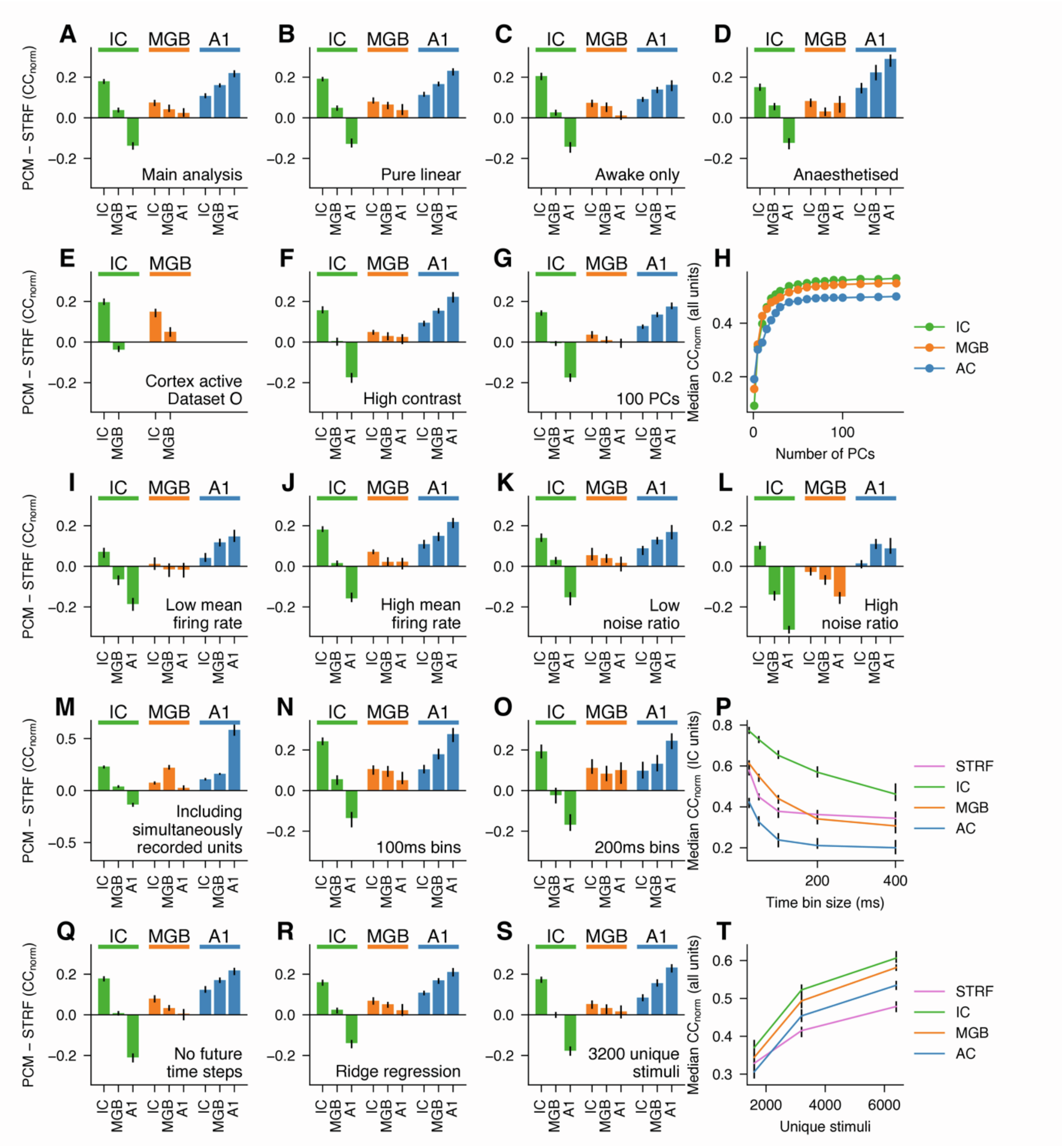


**Figure S5: Control analyses on subsets of data, Related to Figure 5.**
Most panels show the median difference between prediction accuracy ($CC_{norm}$) of population communication models and STRF models for units recorded in the IC, MGB and A1 (target population) with source populations (x-axis) from different processing levels. The values in these panels are comparable to those in **Figure 5D**, but are measured for different variations of the models or datasets. Each panel shows dataset H, unless otherwise stated.

(A) Main results for dataset H, as in **Figure 5D**.

(B) Pure linear STRF and PCM models (no sigmoid output nonlinearity).

(C) Awake data only.

(D) Anesthetized data only.

(E) Dataset O (optogenetic data), including only the condition where cortex was active.

(F) Only high-contrast stimuli were used.

(G) Each source population was reduced to its first 100 Principal Components, and these were used as inputs to the PCM.

(H) PCM performance for target units in IC, MGB and AC when increasing numbers of PCs were used.

(I, J) Only units with firing rate lower (I) or higher (J) than the median were used in the source populations.
(K, L) Only units with noise ratio lower (K) or higher (L) than the median were used in the source populations.
(M) Simultaneously-recorded units were included in the source populations when available (IC to IC, MGB to MGB and A1 to A1 conditions)
(N, O) Time bins of 100ms (N) and 200ms (O) were used.
(P) STRF and PCM (IC source population) performance against time bin size.
(Q) No future (acausal) time steps were included in the PCMs.
(R) Ridge regression was used instead of lasso regularization in the STRFs and PCMs.
(S) 3200 unique stimuli (half of the dataset) were used to train STRFs and PCMs.
(T) STRF and PCM (IC source population) performance when one quarter (1600 stimuli) or half (3200 stimuli) of the unique stimuli were used to train each model.

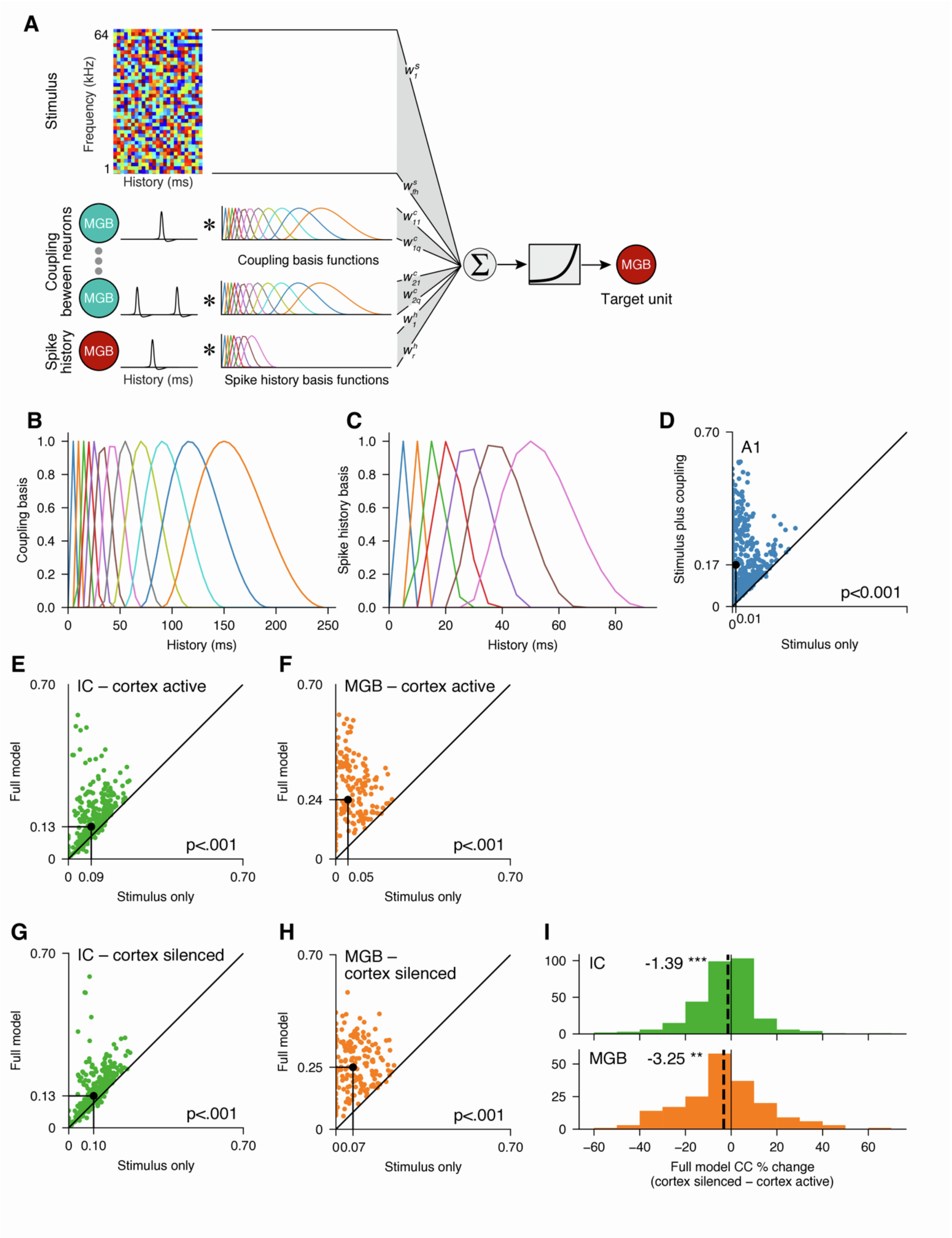


**Figure S6: GLM analysis of neuron-to-neuron communication, Related to Figure 7.**
(A) Schematic showing the full GLM model, which consists of an STRF component (similar to STRF models), with additional regressors describing coupling between simultaneously-recorded neurons and the spike history of the target neuron. The spike coupling regressors are the result of convolving the responses of simultaneously-recorded neurons with basis functions (B) spanning latencies up to 250 ms at 5 ms resolution. The spike history regressors are the result of convolving the target neuron's responses with basis functions (C) spanning latencies up to 90 ms. To fit the neural

responses, weights are estimated for all regressors that minimize the MSE between the model output and the target neuron's responses, using a Poisson GLM[35].
(D) Comparison of coupling model vs stimulus only model for auditory cortex (dataset H; not optogenetic data), similar to Figure 7C,D.
(E-H) The full model, including coupling and spike history, predicts neuronal responses better than the same model including the stimulus only, in both IC (E, G) and MGB (F, H), and both when the cortex was active (E, F) and when it was silenced optogenetically (G, H).
(I) Performance of the full model is slightly higher when the cortex was active than when the cortex was silenced, for both IC and MGB.
